# Bacteriome and resistome dysbiosis in subclinical mastitis and antibiotic-treated milk of dairy cows

**DOI:** 10.1128/mra.01070-25

**Published:** 2025-11-28

**Authors:** M. Nazmul Hoque, M. Shaminur Rahman

**Affiliations:** 1 Molecular Biology and Bioinformatics Laboratory, Department of Gynecology, Obstetrics and Reproductive Health, Gazipur Agricultural University 198780, Gazipur, Bangladesh; 2 Department of Microbiology, Jashore University of Science and Technology 421984, Jessore, Bangladesh; Fluxus Inc., Sunnyvale, California, USA

**Keywords:** dairy cows, subclinical mastitis, antibiotic treatment, bacteriome, resistome, metagenome sequencing

## Abstract

Shotgun metagenomics revealed distinct bacteriome profiles in subclinical mastitis, antibiotic-treated, and healthy cow milk, with enriched resistance repertoires in diseased and treated samples. Findings highlighted the need for better diagnostics, precision antimicrobial use, and antibiotic alternatives to ensure milk safety and address antimicrobial resistance in dairy farming.

## ANNOUNCEMENT

Mastitis remains a major challenge in dairy farming (1), with subclinical mastitis (SCM) posing the greatest burden in Bangladesh due to poor diagnostics and farm practices (2–4). SCM often goes unnoticed, reducing milk yield, impairing fertility, and increasing antibiotic use, antimicrobial resistance, and microbiome dysbiosis in affected cows (5–7). This altered microbiota composition has been associated with persistence of infection and increased presence of antibiotic resistance genes (ARGs) (5, 8, 9). This study employed whole metagenome shotgun (WMS) to reveal bacteriome–resistome profiles in healthy, SCM, and antibiotic-treated milk.

We analyzed 30 milk samples (10 SCM, 10 antibiotics treated milk [ANT], and 10 healthy cow’s milk [HM]; Table 1) collected from 30 lactating crossbred dairy cows on a commercial farm in the Dhaka district, Bangladesh. The cows represented three common breed types, namely Holstein Friesian Cross (HFC, *n* = 11), Sahiwal Cross (SC, *n* = 9), and Local Zebu (LZ, *n* = 10), and were in early to mid-lactation, ranging from 5 to 60 days post-parturition. DNA was extracted with the DNeasy Blood and Tissue Kit (QIAGEN, Germany) on an automated platform following established protocols (7, 10) and quantified with NanoDrop ND-2000 (11). Libraries were prepared with Nextera XT (7, 11, 12), assessed via Qubit 2.0 and Agilent Bioanalyzer 2100, and sequenced (2×150  bp) on the NovaSeq 6000 platform (Illumina Inc., USA). Metagenomic reads were processed using Trimmomatic v0.39 (13), then analyzed via CZ ID (14). Phyloseq R package v4.4 (15) and ggplot2 (16) were used for data processing and visualization. Statistical comparisons employed Kruskal-Wallis tests and SPSS v25.0 (10).

**TABLE 1:** Study sample information, SRA accession numbers of the whole metagenome sequences and OTUs (operational taxonomic units = 679) mapped against bacterial taxa^*a*^

Sl. no.	Sample ID	Sample type	Breed	Lactation stage (Days after parturition)	Milk yield (Liter)	Farm location	No. of raw reads (before trimming)	No. of quality reads (after trimming)	No. of mapped reads	No. of observed OTUs	SRA accessions
1	SCM1	SCM	HFC	45	22	Dhaka	12,113,746	12,060,664	1,875,533	73	SRR28341692
2	SCM2	SCM	HFC	22	14	Dhaka	11,843,987	11,776,824	1,081,465	62	SRR28341686
3	SCM3	SCM	SC	7	7	Dhaka	15,008,310	14,656,341	1,484,361	37	SRR28341675
4	SCM4	SCM	LZ	60	3	Dhaka	12,070,051	12,034,338	559,680	75	SRR28341664
5	SCM5	SCM	LZ	40	4	Dhaka	11,999,831	11,953,257	383,352	90	SRR28341663
6	SCM6	SCM	SC	15	6	Dhaka	8,712,546	8,489,918	278,135	35	SRR28341691
7	SCM7	SCM	LZ	30	5	Dhaka	15,008,310	14,656,341	308,310	39	SRR28341690
8	SCM8	SCM	HFC	7	18	Dhaka	11,920,062	11,896,202	91,360	207	SRR28341689
9	SCM9	SCM	SC	10	12	Dhaka	12,887,239	12,642,929	1,178,180	47	SRR28341688
10	SCM10	SCM	HFC	15	18	Dhaka	19,480,895	19,272,605	251,571	68	SRR28341687
11	ANT1	ANT	HFC	45	22	Dhaka	8,215,147	7,910,230	308,752	157	SRR28341685
12	ANT2	ANT	HFC	22	14	Dhaka	7,122,952	6,854,102	115,384	42	SRR28341684
13	ANT3	ANT	SC	7	7	Dhaka	8,473,979	8,254,757	291,453	54	SRR28341683
14	ANT4	ANT	LZ	60	3	Dhaka	10,910,737	9,753,576	628,299	51	SRR28341681
15	ANT5	ANT	LZ	40	4	Dhaka	12,130,996	12,076,039	142,524	44	SRR28341682
16	ANT6	ANT	SC	15	6	Dhaka	8,015,147	7,810,230	221,500	156	SRR28341680
17	ANT7	ANT	LZ	30	5	Dhaka	7,358,494	7,204,336	1,047,845	323	SRR28341679
18	ANT8	ANT	HFC	7	18	Dhaka	8,031,995	7,829,594	909,767	225	SRR28341678
19	ANT9	ANT	SC	10	12	Dhaka	11,118,015	10,339,228	177,002	131	SRR28341677
20	ANT10	ANT	HFC	15	18	Dhaka	7,799,937	7,572,239	107,620	41	SRR28341676
21	HM1	HM	HFC	22	14	Dhaka	12,096,858	12,066,530	71,227	159	SRR28341674
22	HM2	HM	HFC	10	9	Dhaka	10,097,985	8,884,528	102,098	77	SRR28341673
23	HM3	HM	HFC	35	12	Dhaka	3,301,658	3,249,161	59,822	130	SRR28341672
24	HM4	HM	HFC	5	19	Dhaka	12,259,145	12,221,271	349,108	81	SRR28341670
25	HM5	HM	SC	15	10	Dhaka	11,018,572	10,071,382	50,954	220	SRR28341671
26	HM6	HM	SC	5	8	Dhaka	9,928,322	9,706,011	470,371	129	SRR28341669
27	HM7	HM	SC	45	12	Dhaka	8,480,389	8,260,368	15,713	26	SRR28341668
28	HM8	HM	LZ	55	5	Dhaka	17,227,741	17,037,343	17,364	25	SRR28341667
29	HM9	HM	LZ	40	4	Dhaka	10,373,218	10,119,570	49,263	24	SRR28341666
30	HM10	HM	LZ	24	4	Dhaka	11,988,452	11,960,796	21,577	250	SRR28341665

^*a*^ SCM: Subclinical Mastitis Milk; ANT: Antibiotics treated Milk; HM: Healthy Cows Milk; HFC: Holstein Friesian Cross; SC: Sahiwal Cross; and LZ: Local Zebu.

**Fig 1:**
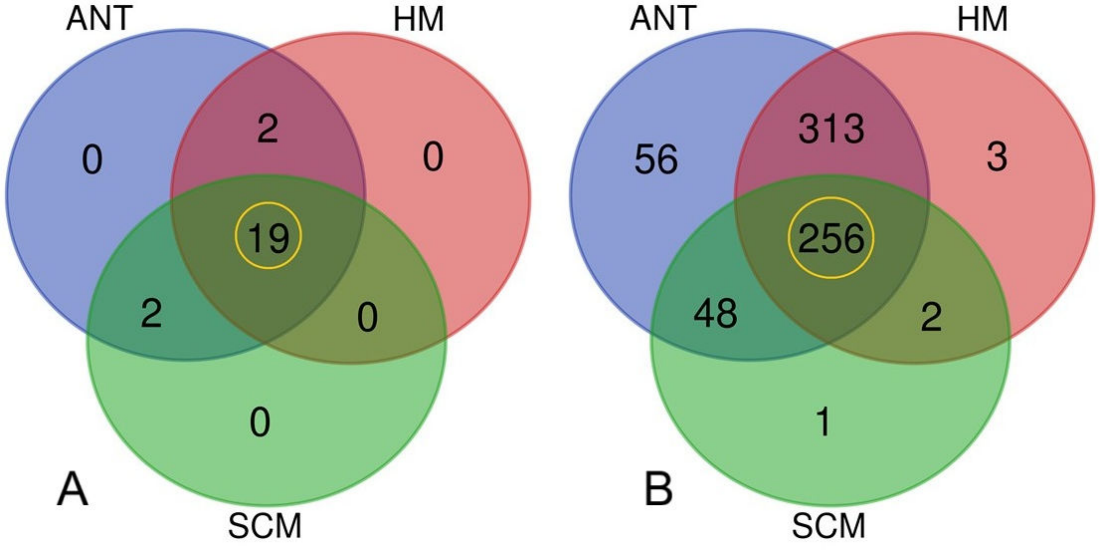
Taxonomic composition of bacteriomes. (**A**) Bacterial phyla detected in SCM milk, ANT, and HM samples. (**B**) Bacterial species identified in SCM, ANT, and HM samples. Shared taxa are highlighted in a yellow circle.

Shotgun WMS of 30 milk samples yielded 318.78 million high-quality reads, which were mapped to 679 microbial taxa (Table 1). Figure 1 depicts the phylum and species level bacteriome composition in the SCM, ANT, and HM samples. At the species level, SCM samples were dominated by *Acinetobacter johnsonii* (39.7%) and *Escherichia coli* (15.0%). In contrast, ANT samples showed enrichment of *Pseudomonas aeruginosa* (1.2%), *P. putida* (2.9%), and *Raoultella ornithinolytica* (2.0%), indicating antibiotic-driven selection. HM samples were largely composed of beneficial species, such as *P. lundensis* (34.7%) and *Actinoalloteichus* spp. (4.4%). ARGs conferring resistance to macrolides (*MLS23S*, ~21.9%), aminoglycosides (*A16S*, ~11.9%), and efflux pumps (*TTGB*, ~3.8%) were significantly enriched in ANT samples (*P* < 0.01). Correlation analysis revealed strong associations between *R. ornithinolytica* and multiple ARGs (r = 0.83), and between *E. coli* and *Enterococcus faecium* (r = 0.63). Notably, *Clostridium botulinum* was prevalent in all groups (22.2%–42.7%), posing potential food safety risks.

## Data Availability

The shotgun whole metagenome sequencing data are available at the NCBI Sequence Read Archive (SRA) under BioProject accession number PRJNA1081607. The accession numbers for all 30 SRA experiments are listed in Table 1.
